# Shear Strength of Hybrid Fibre-Reinforced Ternary Blend Geopolymer Concrete Beams under Flexure

**DOI:** 10.3390/ma14216634

**Published:** 2021-11-03

**Authors:** V. Sathish Kumar, N. Ganesan, P. V. Indira

**Affiliations:** 1 Faculty of Civil Engineering, Architecture and Geodesy, University of Split, 21000 Split, Croatia; 2 Department of Civil Engineering, National Institute of Technology Calicut, Kozhikode 673601, India; ganesan@nitc.ac.in (N.G.); indira@nitc.ac.in (P.V.I.)

**Keywords:** beam, geopolymer concrete, hybrid fibre-reinforced concrete, shear strength, ternary blend

## Abstract

The need to promote sustainable civil infrastructure is one of the most important concerns in the construction industry. Geopolymer composites are one of the promising eco-friendly materials for the development of low carbon concrete. The main objective of this experimental investigation is to study the effect of hybrid fibres on the shear strength of flexural members made with ternary blend geopolymer concrete (TGPC). A total number of 27 reinforced concrete beams of size 100 mm × 150 mm × 1200 mm were cast and tested for shear. M55 grade of concrete was considered in this study. Crimped steel fibres and polypropylene fibres with an aspect ratio of 66 and 300, respectively, were used in this work. The main variables considered in this investigation involve two volume proportions of steel fibres, viz., 0.5% and 1% as well as four volume proportions of polypropylene fibres viz., 0.1%, 0.15%, 0.2% and 0.25%. The hybrid fibre-reinforced ternary blend geopolymer concrete (HTGPC) beams were compared with TGPC beams without fibres. From the test results, it was clear that incorporating hybrid fibres improved the shear strength and changed the type of failure of the beam from shear to flexure. Moreover, a method to predict the ultimate shear strength of HTGPC was proposed, and the estimated values were found to be the same as the test results.

## 1. Introduction

Geopolymer concrete (GPC) is a new environmentally friendly material that reduces the global warming of the Earth and the greenhouse effect by replacing ordinary Portland cement (OPC). Portland cement is not considered a sustainable material due to the emission of a substantial amount of carbon dioxide (CO_2_) into the atmosphere during its production [1,2,3]. GPC developed by Davidovits provides a suitable replacement for conventional concrete [4,5]. GPC is produced from an aluminosilicate source material using alkaline activators. It reduces the CO_2_ footprint and effectively utilises industrial wastes such as silica fumes, fly ash, ground granulated blast furnace slag (GGBS), etc. GPC has excellent mechanical properties and better fire resistance than conventional cement concrete. It reduces the carbon footprint and saves water for curing since the strength of GPC is from the polymerisation process, while the strength of OPC is due to the hydration of cement [6]. Ternary blend geopolymer concrete (TGPC) is developed by mixing three different source materials of varying particle sizes to form a denser concrete, resulting in improved properties compared to GPC [7,8]. Many researchers, aiming to provide knowledge about the effect of such concrete as structural elements, have discussed the structural behaviour of reinforced GPC members. Sofi et al. [9] studied the bond behaviour of geopolymer concrete with a steel bar by performing the direct pull-out test and the beam-end test. They concluded that the development length for GPC could be predicted using the equations provided in the standards for normal concrete. Sumajouw et al. [10] studied the effect of tensile reinforcement ratio on the behaviour of reinforced GPC and reported that the flexural capacity and ductility index are similar to conventional cement concrete. Many other studies also highlighted that the GPC beams performed similarly to conventional reinforced concrete under flexure [11,12,13,14].

A lot of research has been performed to explore the effect of fibres on the mechanical properties of concrete [15,16,17]. Naaman and Reinhardt [18] reported that adding a little fraction of short Torex fibres, which are engineered to achieve optimal properties in the order of 0.5% to 1% by volume, in concrete could enhance the toughness in the post-cracking response by bridging across the cracks. In the hybrid fibres system, the short fibres can bridge across the microcracks while the long fibres are more effective at controlling the progression of macrocracks [19,20,21,22]. The advantages of having a combination of metallic and non-metallic fibres in cement concrete are improving the mechanical properties and reducing the total cost of the concrete [23,24,25]. Navid et al. [26] studied the fibre–matrix interaction of fly ash-based GPC incorporated with steel and polypropylene fibres. They reported that the fibres played a vital role in the interfacial bond and improved properties such as energy absorption and flexural strength. Attempting to improve the tensile behaviour of high strength concrete has recently been gaining importance among researchers. High-performance fibre-reinforced concrete is a recently developed concrete that possesses high strength and ductility for use in structures where bending is dominant. Doo-Yeol et al. [27] investigated the effect of fibre length and placement method on the flexural behaviour of ultra-high-performance fibre-reinforced concrete (UHPFRC). They reported that the fibres placed in the centre exhibited higher flexural strength since more fibres existed in the crack plane. The material and bond properties of UHPFRC with micro steel fibres were reported by Doo-Yeol et al. [28]. They found that the CMR model was appropriate for predicting the ascending portion of the bond stress–slip response between the UHPFRC and the steel rebar. Umberto et al. [29] developed a numerical model for tracing the structural response of steel bar-reinforced UHPFRC enhanced with nanomaterials.

Several studies were carried out in the past on fibre-reinforced concrete beams without stirrups for normal concrete, high strength and high-performance concrete (HPC), and the studies proved the efficiency of the fibres in increasing the shear strength [30,31]. Victor et al. [32] examined the ultimate shear strength of fibre-reinforced mortar and concrete beams without stirrups and noted that the fibres prevented the shear failure. Many authors proposed empirical equations for predicting the ultimate shear strength for fibre-reinforced cement concrete [30,33,34,35]. Ambily et al. [36] and Ganesan et al. [37] reported the response of geopolymer concrete beams tested under shear and the effect of steel fibres on the shear strength of beams. All these works are limited to normal concrete, HPC, UHPFRC and fly ash-based GPC composites. However, studies on the behaviour of hybrid fibre-reinforced ternary blend geopolymer concrete (HTGPC) beams under shear are not yet reported. Hence, in this study, an attempt is made to analyse the shear strength of TGPC due to the influence of hybrid fibres.

## 2. Experimental Programme

### 2.1. Materials

The primary source material for the TGPC used in this work was low-calcium class F fly ash procured from Mettur Thermal Power Station in Tamil Nadu, India, conforming to the requirements of IS 3812:2003 [38]. Its chemical composition includes Al_2_O_3_ (27.75%) and SiO_2_ (55.36%). It is a dark grey powder and has a specific gravity of 2.30. The average size of the fly ash particle is 75 microns. GGBS consisting of 77.37% of total CaO, MgO and SiO_2_ conforming to BS 6699:1992 [39] was also used as one of the source materials. The colour of GGBS is off-white and has a specific gravity of 2.88. The mean particle size of GGBS is 30 microns. Metakaolin procured from the local supplier was used as a third precursor for the TGPC. It has a specific gravity of 2.56 with a creamish-ivory powder appearance. It is composed of 92% of combined SiO_2_, Al_2_O_3_ and Fe_2_O_3_ elements. The average particle size of metakaolin is 2–3 microns. These three materials were combined to form a ternary binder for the TGPC. The detailed properties of the source materials and their morphological information were provided in the previous study performed by the authors [7,40].

**Figure 1:**
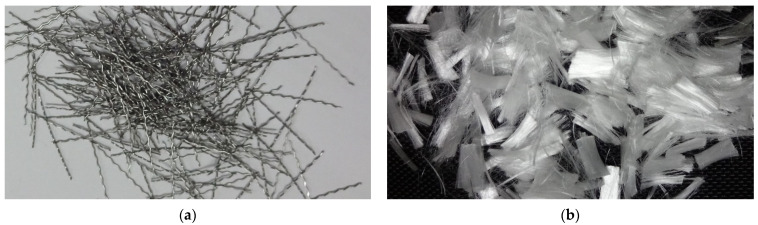
Fibres used: (**a**) crimped steel fibres; (**b**) polypropylene fibres.

Locally available crushed stone (M-Sand) passing through a 4.75 mm (No.4) IS sieve conforming to Zone II of IS 383:1970 (reaffirmed 2002) [41] with a fineness modulus and specific gravity of 2.92 and 2.39 was used as fine aggregate. Coarse aggregate with a maximum size of 12.5 mm with a fineness modulus and specific gravity of 6.92 and 2.78 was also used. A blend of sodium hydroxide (NaOH) in pellet form with 99% purity and a sodium silicate (Na_2_SiO_3_) solution consisting of 8% of Na_2_O, 28% of SiO_2_ and 64% of water by mass was used as an alkaline activator. Naphthalene-based water reducing admixture, Conplast SP 430, was added for better workability. The hybrid fibres used in this study consist of (i) crimped steel fibres with a length of 30 mm and 0.45 mm diameter and (ii) polypropylene fibres having a length of 12 mm and 40 micron diameter. Figure 1 shows images of the steel and polypropylene fibres added in the mixture. The ultimate tensile strength of the steel and polypropylene fibres was 800 MPa and 600 MPa, respectively.

### 2.2. Mix Proportions for TGPC

As standard mix design is not available for geopolymer concrete, the TGPC mix proportion for M55 concrete was arrived at by trial and error based on Rangan’s recommendations [42]. The proportion of fly ash, GGBS and metakaolin the alkaline activator-to-binder ratio and the molarity of NaOH were obtained from the detailed studies carried out by the authors, which are presented elsewhere [40,43] and are used in the present experimental investigation. Thus, the TGPC consists of 60% fly ash, 25% GGBS and 15% metakaolin. Moreover, the alkaline activator-to-binder ratio was selected as 0.3, and the molarity of NaOH was maintained as 14 M. The water-to-binder ratio was kept constant at 0.2. Superplasticiser was added to the mix by 1.5% of the binder’s total weight for better workability. The summary of the TGPC mix proportion is provided in Table 1. The fibres were added to the same mix proportion at different levels to study their effect on the concrete.

### 2.3. Specimen Details

**Figure 2:**
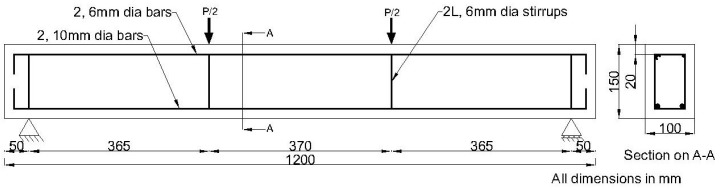
Reinforcement details of the specimen.

The shear behaviour of HTGPC beams under flexure was studied by casting and testing 27 reinforced concrete beams of size 100 × 150 × 1200 mm^3^ with different volume fractions of fibres. Three specimens were tested for each combination of fibres and the average of the results was taken for analysis. The details of reinforcement of the specimen and the dimensions are provided in Figure 2. Two nos. of 10 mm diameter high yield strength deformed (HYSD) bars and two nos. of 6 mm diameter bars were provided at the bottom and top of the beam, respectively. The properties of the reinforcing bars are given in Table 2. Two-legged stirrups were provided using a 6 mm diameter bar at the loading and supporting points. No shear reinforcement was provided in the shear span to ensure shear failure [37].

### 2.4. Mixing, Casting and Curing Procedure

The test specimens were prepared using the dry materials in the horizontal drum-type concrete mixture, including ternary source material, coarse aggregates and fine aggregates. The NaOH pellets were mixed with water to prepare a 14 M solution, and then a Na_2_SiO_3_ solution was added to it to form the alkaline activator 24 h before casting. The ratio of Na_2_SiO_3_ to NaOH by weight was kept constant at 2.5:1. While preparing the dry mixture, polypropylene fibres were added to the mix for the uniform distribution of fibres. Superplasticiser, alkaline activator and water were added to the dry mix, followed by the steel fibres. The reinforcement cage was kept inside the steel mould; the mixture was then poured in three layers into the mould and vibrated using a needle vibrator for better compaction. The top surface was smoothened using a trowel and covered with plastic film to avoid moisture loss while curing. After 24 h of rest, the beams were cured at 60 °C for the next 24 h inside the steam-curing chamber. The samples were left at room temperature until testing.

### 2.5. Testing

**Figure 3:**
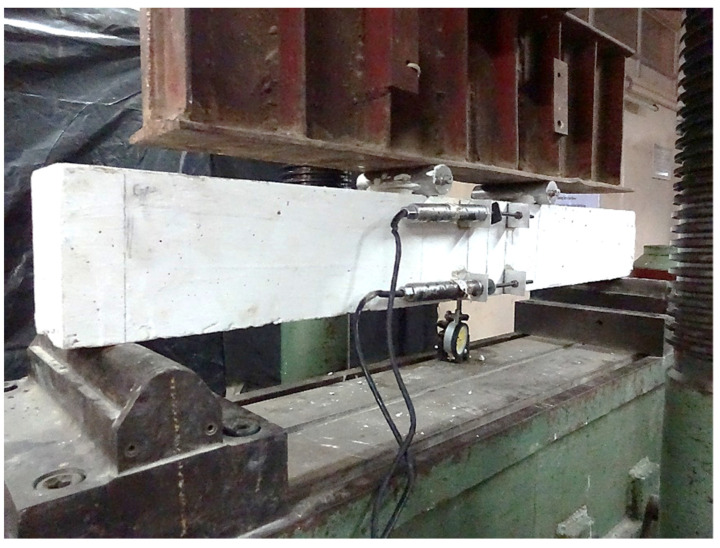
Actual test setup.

The beams were whitewashed and then tested after 28 days under a four-point loading method to observe the crack patterns. The beams were tested in a compression and bending testing machine (GDR, Blue Star Engg. Co. Pvt. Ltd., Madras, India) of 3000 kN capacity at a constant loading rate of 4 kN/min until failure. The test was conducted in a load-controlled test setup and necessary precautions were taken to keep a constant loading rate. The deformations were observed by using electrical gadgets such as LVDTs, so that at every stage of application of loading the time taken for the observation was minimal. Two LVDTs with a least count of 1 micron were fixed at the top and bottom of the mid-span. The deformation at the mid-span was noted using a dial gauge having the least count of 0.01 mm and 25 mm travel. A crack width microscope with the least count of 0.02 mm was used to observe the cracks developed in the specimens. The readings were taken at each load increment of 2 kN during the test. The actual test setup is shown in Figure 3.

## 3. Results and Discussions

The test results of the beams are shown in Table 3. The values given in the table are the average of three identical beams tested with the same fibre content and loading condition.

### 3.1. Load–Deformation Characteristics

**Figure 4:**
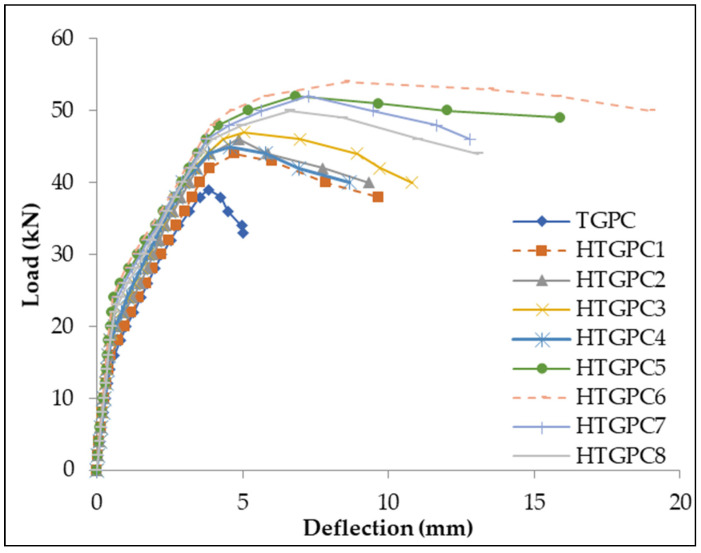
Load versus deflection plots.

The load–deflection curves for TGPC and HTGPC beams are shown in Figure 4. It can be noted that the linearity of the curves for all the specimens deviates after the first crack load. After the first crack load, multiple cracks were formed and the slope of the curve reduced and became nonlinear [44]. The HTGPC beams show a more or less flat portion of the curve beyond the ultimate load. In contrast, a sudden fall in the load was observed after the ultimate load for the TGPC beams without hybrid fibres. From the figure, it can be observed that the first stage was linear up to the formation of the first crack. After the initial crack, the beams underwent post-cracked behaviour and the load–deformation curve behaved nonlinearly until the peak load. The initiation of inclined cracks was influenced by strain–softening of tensile concrete, but in the progressive development of diagonal cracks the dowel action of steel bars and the fibres appeared to predominate [45]. It can also be noted from the figure that the HTGPC beams with 0.5% steel fibres exhibited improved deformation with the addition of polypropylene fibres up to 0.2%. In comparison, the deformation of HTGPC beams with 1% steel fibres improved up to 0.15% polypropylene fibres. It can be observed that the further addition of polypropylene fibres resulted in reduced deformation; this is due to poor workability in the TGPC mixture with a high content of hybrid fibres [46]. In contrast, low hybrid fibre content resulted in only a marginal improvement in the behaviour of the HTGPC specimens with 0.5% steel fibres [47,48]. HTGPC beams with higher fibre proportion show less deformation for the same magnitude of the load, which shows the ductility increases due to the incorporation of hybrid fibres. HTGPC beams with a mix of 1% steel and 0.15% polypropylene fibre were found to perform better than all other specimens.

### 3.2. Cracking Behaviour and Failure Modes

**Figure 5:**
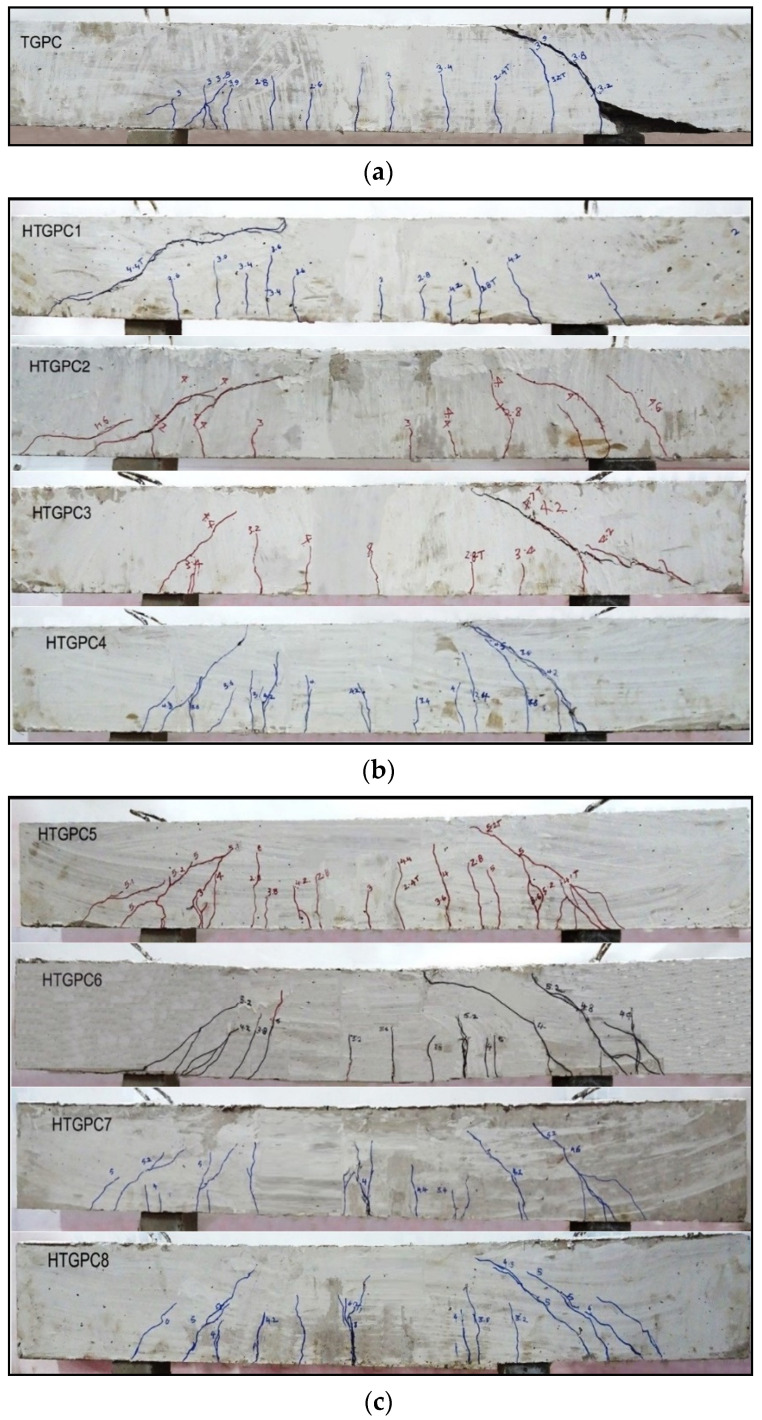
Crack pattern of typical tested beams: (**a**) TGPC beam; (**b**) HTGPC beams with 0.5% steel fibres; (**c**) HTGPC beams with 1% steel fibres.

Figure 5 shows the crack patterns of the typical tested specimens after failure. Figure 5a shows that, in the plain TGPC beam, the first crack was initiated in the flexural span. When the load increased, diagonal cracks developed in the shear span and additional flexural cracks formed. As the load increased further, the existing cracks widened; at the final stage, the diagonal cracks developed faster, resulting in the beam’s failure [49]. In the TGPC specimen, the failure occurred at the shear span when one of the diagonal cracks widened and reached the specimen’s top. It can also be seen that, in a plain TGPC beam, the spalling of cover concrete occurred [50]. The addition of hybrid fibres in TGPC resulted in many finer cracks compared with specimens without fibres. In HTGPC specimens, the cracks were developed and widened at higher loads [51]. However, at corresponding loads, the crack widths of HTGPC specimens with 1% steel fibres were 60% lower than the cracks formed in TGPC specimens, whereas the average crack width was 55% lower for the specimens with 0.5% steel fibres. It may also be noted that, in HTGPC specimens, the flexural cracks are more predominant than the cracks in the shear span. In general, the incorporation of hybrid fibres delayed the first crack, increased the shear strength and changed the failure mode from shear to flexure [44].

### 3.3. Ultimate Shear Strength

Table 3 shows the test results of the tested specimens. The beams with hybrid fibres show improved ultimate shear strength when compared with TGPC beams without fibres [52]. The ultimate shear strength for HTGPC beams with 1% steel and 0.15% polypropylene fibre increased by 38.46% compared with TGPC beams. This may be due to the prevention and deviation of cracks from their path by the fibres bridging across the cracks. Hence, for further propagation of cracks, the beams with fibres need more energy, which consecutively improves the beam’s load-carrying capacity.

## 4. Design Equations Available in the Literature for Shear Strength of Reinforced Concrete Flexural Members

### 4.1. Ashour et al.

Ashour et al. [33] proposed an equation by considering various factors such as steel fibre content, shear span-to-depth ratio and longitudinal steel ratio to predict the shear strength of fibre-reinforced concrete beams.

- Modification of ACI building code equation:

The shear strength equation obtained by modifying the ACI Building Code equation is given by,

(1) $$V_{u}=\left(0.7\sqrt{{f^{\prime }}_{c}}+7F\right)\;\frac{d}{a}+17.2\rho \frac{d}{a}$$

- Modification of Zutty’s equation:

Zutty’s equation was modified by the factor F to account for fibres, and it is given as,

For *a/d* > 2.5,

(2) $$V_{u}=\left(2.11\sqrt{{f^{\prime }}_{c}}+7F\right)\;{\left(\rho \;d/a\right)}^{0.333}$$

For *a/d* > 2.5,

(3) $$V_{u}=\left[\left(2.11\sqrt[{3}]{{f^{\prime }}_{c}}+7F\right){\left(\rho \frac{d}{a}\right)}^{0.333}\right]\left[\frac{2.5\;d}{a}+V_{b}\;\left(2.5-\frac{d}{a}\right)\right]$$

### 4.2. Kwak et al.

Kwak et al. [34] considered different volume fractions of fibres and different shear span-to-depth ratio; the following equation was proposed to obtain the shear strength,

(4) $$V_{u}=3.7\;e\;f_{ct}{}^{2/3}\;{\left(\rho \frac{d}{a}\right)}^{1/3}+0.8V_{b}$$

where:

*e* = 1.0 for *a/d* > 3.4 and 3.4 *d/a* for *a/d* ≤ 3.4

### 4.3. Li et al.

Taking into account split tensile and flexural strength, as well as reinforcement ratio and shear span-to-depth ratio, the following equation was proposed by Li et al. [32] to determine the shear strength,

For *a/d* > 2.5,

(5) $$V_{u}=1.25+4.68\;\left[{\left(f_{f}f_{ct}\right)}^{3/4}\;{\left(\rho \frac{d}{a}\right)}^{1/3}\;{\left(d\right)}^{-1/3}\right]$$

For *a/d* > 2.5,

(6) $$V_{u}=9.16\left[f_{ct}{}^{2/3}\;\rho^{1/3}\left(\;\frac{d}{a}\;\right)\right]$$

### 4.4. Narayanan and Darwish

The equation developed for determining the shear strength of fibre-reinforced beam by Narayanan and Darwish [30] is given as,

(7) $$V_{u}=e\;\left(0.24\;f_{ct}+80\;\rho \frac{d}{a}\;\right)+V_{b}$$

where:

*e* = 1.0 for *a/d* > 2.8 and 2.8 *d/a* for *a/d* < 2.8

### 4.5. Sharma

Sharma [35] conducted tests with different types of fibres and proposed a model for predicting shear strength,

(8) $$V_{u}=\frac{2}{3}\;f_{ct}\;{\left(\frac{d}{a}\right)}^{0.25}$$

The shear strength of the reinforced concrete beams with fibres can be obtained through two design approaches. The first approach uses an empirical equation developed to calculate the contribution of concrete and fibres to the shear strength of the beam. The second approach is more deterministic, where the contribution of fibres to shear strength is computed separately by toughness enhancement in flexure [53]. In this study, the first approach is adopted to calculate the shear strength of the TGPC beams with fibres. Table 4 shows the comparison of experimental shear strength (*V_u_*_(exp)_) and theoretical shear strength (*V_u_*_(the)_) values obtained from the literature. The mechanical properties of HTGPC with different volume fractions of hybrid fibres were obtained from the authors’ detailed experimental work, which is presented elsewhere [7] and used in this present work. It can be observed that there is a variation between the practical and theoretical models in the literature. This inconsistency is due to the fact that all the equations available in the literature were obtained for conventional fibre-reinforced concrete. Therefore, an attempt was performed to modify one of the available equations, giving the low coefficient of variation for the ratio *V_u_*_(exp)_/*V_u_*_(the)_. From the given equations, it is noted that the equation predicted by Li et al. [32] gives the low value of the coefficient of variation (4.76%) and a mean value near 1.0 (0.96). Since different variables such as aspect ratio of fibres, volumetric fraction of fibres and strength of concrete affect the test results, an attempt is made to introduce a factor that accounts for the above parameters.

**Figure 6:**
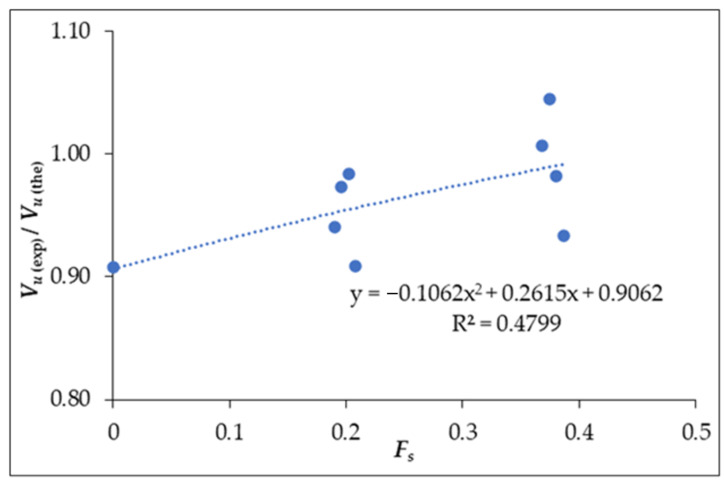
Correction factor (*F_s_*) against *V_u_*_(exp)/_*V_u_*_(the)_.

Hence, the equation of Li et al. [32] was modified further by introducing a factor (*F_s_*) that represents the combination of the above parameters. The equation for the correction factor (*F_s_*) is given below,

(9) $$F_{s}=A_{fs}V_{s}d_{s}\eta_{bs}+A_{fp}V_{p}d_{p}\eta_{bp}$$

where *η_bs_* and *η_bp_* are the bond efficiency factor taken as 1.2 for crimped steel fibres and 1.0 for round straight fibres, respectively [54]. From the calculated shear strength values using Equation (5), the ratios of *V_u_*_(exp)/_*V_u_*_(the)_ were obtained and related to *Fs* as given in Figure 6. The regression equation from the plot is,

*V_u_*_(exp)_/*V_u_*_(the)_ = −0.1062(*F_s_*)^2^ + 0.2615(*F_s_*) + 0.9062 (10)

To predict an equation for obtaining the ultimate shear strength of HTGPC beams (*V_u_*_(pre)_), Equation (10) is modified by replacing *V_u_*_(exp)_ with *V_u_*_(pre)_. Thus, the modified equation for predicting the shear strength of HTGPC is obtained as,

*V_u_*_(pre)_ = *V_u_*_(the)_ (−0.1062(*F_s_*)^2^ + 0.2615(*F_s_*) + 0.9062) (11)

by substituting Equation (5) in the above Equation (11),

(12) $$V_{\mathrm{u(pre)}}=1.25+4.68\left[{\left(f_{f}f_{ct}\right)}^{3/4}{\left(\rho \frac{d}{a}\right)}^{1/3}{\left(d\right)}^{-1/3}\right]\;(-0.1062(F_{s}{)}^{2}+0.2615(F_{s})+0.9062)$$

**Figure 7:**
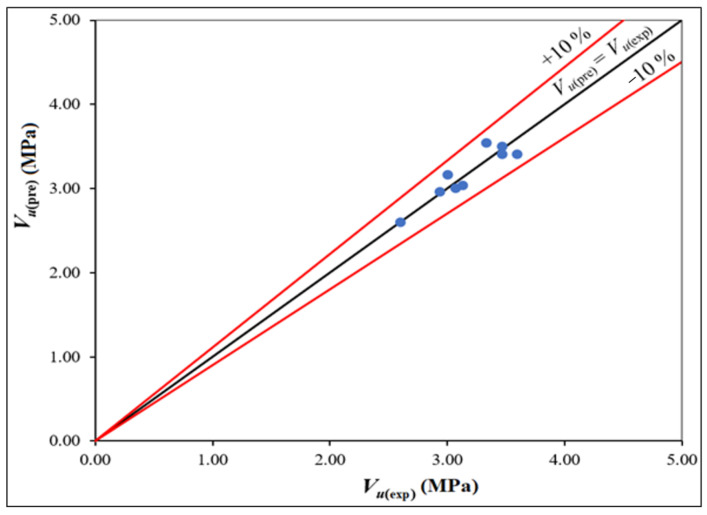
Comparison of experimental and predicted values of shear strength.

From Equation (12), the ultimate shear strength for all the beams was calculated and the results were compared with the experimental values. *V_u_*_(pre)_ versus *V_u_*_(exp)_ values were plotted and are shown in Figure 7. It can be noted from Figure 7 that most of the points are lying near the line of equality and well inside the ±10% lines of agreement. The coefficient of variation and *V_u_*_(pre)/_*V_u_*_(exp)_ mean value are 3.70% and 1.0. This shows that the predicted equation is found to correlate convincingly with the experimental test results. The experimental investigations note that HTGPC is an important alternative to cement concrete composites, which will lead to the development of sustainable composites. It can also be noted that the shear strength of the concrete can be improved, and the shear failure in reinforced concrete beams can be avoided with the incorporation of steel and polypropylene fibres.

## 5. Conclusions

The following conclusions may be derived based on this experimental investigation into the shear strength of hybrid fibre-reinforced ternary blend geopolymer concrete beams under flexure:

1. The addition of hybrid fibres in TGPC beams modified the cracking pattern and failure from shear to flexure.
2. Due to hybrid fibres, the first crack load and ultimate shear strength of HTGPC beams were improved by up to 85% and 38.5%, respectively, compared to the TGPC beams.
3. HTGPC specimens with a combination of 1% steel fibres and 0.15% polypropylene fibres showed better results and suffered higher deflections, indicating a relative increase in ductility compared with other specimens.
4. A method for predicting the ultimate shear strength for HTGPC was proposed to account for the effect of hybrid fibres in TGPC. The predicted values of the ultimate shear strength were found to compare convincingly with the experimental test results.
5. The modified equation for predicting the shear strength of HTGPC beams limits up to 1% steel and 0.25% polypropylene fibres. The obtained test results will be helpful in the rational design of HTGPC beams.

## Figures and Tables

**Table 1:** Mix proportion of ternary blend geopolymer concrete.

Materials	Quantity, kg/m^3^
Fly ash	237.47
GGBS	122.61
Metakaolin	64.53
Coarse aggregate	1293.60
Fine aggregate	554.40
NaOH solution	36.40
Na_2_SiO_3_	90.99
Superplasticizer	6.37
Water	84.92

**Table 2:** Properties of steel bars.

Nominal Diameter of Bar, mm	Actual Diameter of Bar, Mm	Yield Strength, MPa	Ultimate Strength, MPa	Modulus of Elasticity, GPa
10	9.97	534	584	244
6	6.09	526	578	232

**Table 3:** Test results.

Specimen	Volume Fraction, %		Compressive Strength, MPa	Split Tensile Strength, MPa	Flexural Strength, MPa	First Crack Load, kN	Ultimate Load, kN	Deflection at Ultimate Load, mm	Ultimate Shear Strength, MPa
	*V_s_*	*V_p_*							
TGPC	0	0	57.23	4.72	5.62	14	39	3.82	2.60
HTGPC1	0.5	0.1	61.47	6.00	6.48	16	44	4.74	2.93
HTGPC2		0.15	61.77	6.12	6.52	18	46	4.86	3.07
HTGPC3		0.2	61.21	6.25	6.54	19	47	5.04	3.13
HTGPC4		0.25	62.23	6.37	6.58	18	45	4.56	3.00
HTGPC5	1	0.1	66.93	6.27	7.76	25	52	6.82	3.47
HTGPC6		0.15	65.77	6.32	7.85	26	54	8.52	3.60
HTGPC7		0.2	64.09	6.48	7.80	24	53	7.26	3.47
HTGPC8		0.25	64.80	6.56	7.71	23	50	6.63	3.33

**Table 4:** Comparison of experimental values with theoretical values obtained from the literature.

Specimen	*V_u_*_(exp)_, MPa	*V_u_*_(the)_, MPa						*V_u(_*_exp_ *_)_* */V_u(_*_the_ *_)_*					
		Ashour et al.-I	Ashour et al.-II	Kwak et al.	Li et al.	Narayanan and Darwish	Sharma	Ratio	Ratio	Ratio	Ratio	Ratio	Ratio
	i	ii	iii	iv	v	vi	vii	i/ii	i/iii	i/iv	i/v	i/vi	i/vii
TGPC	2.60	1.62	1.23	1.76	2.86	1.38	2.17	1.60	2.12	1.47	0.91	1.89	1.20
HTGPC1	2.93	2.58	1.71	2.55	3.12	2.29	2.54	1.14	1.72	1.15	0.94	1.28	1.15
HTGPC2	3.07	2.76	1.80	2.69	3.15	2.45	2.59	1.11	1.71	1.14	0.97	1.25	1.18
HTGPC3	3.13	2.92	1.88	2.83	3.18	2.63	2.67	1.07	1.67	1.11	0.98	1.19	1.17
HTGPC4	3.00	3.10	1.97	3.04	3.30	2.86	2.86	0.97	1.52	0.99	0.91	1.05	1.05
HTGPC5	3.47	3.22	2.03	3.15	3.44	2.97	3.02	1.08	1.71	1.10	1.01	1.17	1.15
HTGPC6	3.60	3.37	2.11	3.28	3.45	3.12	3.05	1.07	1.71	1.10	1.04	1.15	1.18
HTGPC7	3.47	3.52	2.18	3.48	3.53	3.36	3.25	0.98	1.59	1.00	0.98	1.03	1.07
HTGPC8	3.33	3.70	2.27	3.62	3.57	3.52	3.30	0.90	1.47	0.92	0.93	0.95	1.01
Average								1.10	1.69	1.11	0.96	1.22	1.13
Coefficient of variation (%)								18.4	10.9	14.2	4.76	22.4	6.14

## Data Availability

Not applicable.

## Nomenclature

*ρ*	percentage of reinforcement in tension
*η_bs_*	bond factor for steel fibre
*η_bp_*	bond factor for polypropylene fibre
*a*	shear span
*d*	effective depth of beam
*d_p_*	diameter of polypropylene fibre
*d_s_*	diameter of steel fibre
*e*	arch action factor
*f_ct_*	tensile strength of concrete
*f’_c_*	cylinder compressive strength
*f_f_*	flexural strength of concrete
*A_fs_*	aspect ratio of steel fibre
*A_fp_*	aspect ratio of polypropylene fibre
*F_s_*	fibre factor
*V_s_*	volume fraction of steel fibres
*V_p_*	volume fraction of polypropylene fibres
*V_u_*	ultimate shear strength
